# Dietary Diversity Practice and Associated Factors among Children Aged 6–23 Months in Robe Town, Bale Zone, Ethiopia

**DOI:** 10.1155/2020/9190458

**Published:** 2020-06-28

**Authors:** Shumi Bedada Damtie, Tomas Benti Tefera, Mekonnen Tegegne Haile

**Affiliations:** ^1^Disease Control and Prevention Department, Bale Zone Health Office, Robe, Ethiopia; ^2^Department of Public Health, Madda Walabu University Goba Referral Hospital, Goba, Ethiopia

## Abstract

**Background:**

Diet diversification is essential to prepare adequate food that is useful for children's physical and cognitive development. Despite the limited studies performed in different parts of Ethiopia, the information about the feeding practice of children in the current study area is not documented. Thus, this study intended to assess the dietary diversity practices and associated factors among children aged 6–23 months.

**Methods:**

Community-based cross-sectional study was conducted on 517 children aged 6–23 months paired with their mothers in Robe town. Systematic sampling technique was applied to select a child-mother pair. Data were collected using a pretested and structured questionnaire. Multivariate logistic regression analyses were used to identify the factors associated with the dependent variable. Adjusted odds ratios with 95% confidence interval were used to assess the strength of association and level of significance.

**Results:**

From a total of 508 children included, making a 98% response rate, 77% of them did not meet the minimum dietary diversity. Children aged 12–23 months were more likely fed diversified food when compared with those aged 6–11 months (AOR = 2.99). Mothers whose educational level was secondary and above (AOR = 3.21), had media exposure (AOR = 3.99), and were knowledgeable about diet diversification (AOR = 8.5) were more likely to feed their child diversified food than their counterpart. Children whose father was a merchant were more likely to receive a diversified diet compared to those whose fathers were daily laborers.

**Conclusions:**

Inadequate practices of minimum dietary diversity observed in the current study area were mainly associated with the child's age, maternal education, mothers' knowledge on diet diversification, and media exposure. Improving knowledge of mothers, increasing their education, and promoting appropriate infant and child feeding practices through media are an important intervention to improve dietary diversity practices.

## 1. Background

Interventions for improving nutrition must focus on the period from conception through the first two years of life. Undernutrition during this period can have an impact on physical growth and brain development that is extensive and largely irreversible [1]. Stunting and other forms of undernutrition reduce a child's chance of survival and hinders optimal health and growth [2]. Its consequences extend to adulthood, increased risk of poor pregnancy outcomes, impaired cognition that results in poor school performance, reduced economic productivity and earnings, and future risk for overweight and subsequently noncommunicable diseases such as hypertension and cardiovascular disease. This, in turn, affects the developmental potential of the country [3]. Child undernutrition is caused by numerous and multidimensional factors; however, lack of diversified or adequate food is the major factor for the problem [4, 5].

Dietary diversity is the number of different food groups consumed over a specified time period [6]. This number of food groups consumed is positively correlated with adequate micronutrient density of complementary foods [7, 8]. Studies indicated that consumption of a diverse diet is important in the reduction of undernutrition among infants and young children aged 6 to 23 months [4, 5, 9–11]. The World Health Organization (WHO) identified seven food groups which provide sufficient energy, protein, and micronutrients for infants and young children aged 6 to 23 months. It recommends children to consume four or more food groups from the seven food groups daily [12, 13].

Nevertheless, meeting the minimum dietary diversity standard has been a major challenge in developing countries including Ethiopia. Recent researches conducted locally and nationally found that the dietary diversity feeding practice in Ethiopia is very poor [14–18]. According to the recent Ethiopian Demographic and Health Survey (EDHS), only 7% of children in Ethiopia were fed minimum acceptable diet [18]. Even though efforts have been made to increase dietary diversity feeding practice in Ethiopia, the country ranked the lowest in East African countries [19]. Surveys reported that urban children indicate better practice than the rural ones. However, the majority of children in both areas still far from meeting the minimum dietary diversity standard as specified by the WHO [12, 20, 21].

A number of studies examined the factors associated with minimum dietary diversity for children aged 6–23 months. Studies reported that maternal education [11, 14, 19, 22–24], age of the child in months [16, 19, 24, 25], socioeconomic status [14, 19, 22], occupation of mothers [15], father's educational status [23, 25], exposure to media [16, 22], number of under-five children in the household [14], child status of breastfeeding [24], women's empowerment, household food security, fruit and vegetable cultivation, and land ownership [26] are factors associated with minimum dietary diversity.

The current study intended to determine the dietary diversity practices and identify associated factors among children aged 6–23 months in the study area. The finding of this study could suggest some of the possible solutions be taken and appropriate strategies to tackle the problems identified for organizations working on children's health and nutrition. Moreover, this work adds to the field of infant and young child feeding, and the results may help public health policies.

## 2. Methods

### 2.1. Study Design, Area, and Period

A community-based cross-sectional study was conducted in Robe, more commonly known as Bale Robe, which is a town in South East Ethiopia located in the Bale Zone of the Oromia Regional State. It is located about 430 kilometers from Ethiopia's capital, Addis Ababa. Bale highland (in which the town located) is highly suitable for agriculture and is known for potential production of wheat and barley in Ethiopia. Robe town has a total of three kebeles and has a total population of 67,124, of which the total of children aged 6–23 months is estimated to be 2,873. In the town, there are two governmental health institutions: one district hospital and one health center. In addition, there are a total of 51 private health institutions (clinics and drug stores) delivering health services in the town (source: Robe town health office 2017). The main market day is Thursday, with other smaller markets being on Tuesdays and Sundays at another place in the town. A study was conducted from March 28 to April 30, 2017.

### 2.2. Source and Study Population

The source populations of the study were all infants and young children aged 6–23 months paired with their mothers who live in the town. Sampled children aged 6–23 months paired with their mothers, who met the inclusion criteria, were the study population.

### 2.3. Inclusion and Exclusion Criteria

Mothers or caregivers of infants and young children aged 6–23 months who were permanent residents (residing in the town for the last six months) were included in the study. The following were excluded from the study: mothers or caregivers who have a health problem that can affect the interview process and households that had a special ceremony on the day prior to data collection.

### 2.4. Sample Size and Sampling Techniques

Sample size was determined by using single population proportion formula. The expected proportion of children practicing minimum dietary diversity (12.6%) was taken from the study conducted in the town from the northern part of Ethiopia [16]. *n*=(*Z*^2^*pq*/*d*^2^), total sample size required (*N*) = *n* + non respondents. Assuming a 95% level of confidence, 3% acceptable margin of error, and 10% possibility for nonresponse rate, then, finally, the minimum sample size of 517 was obtained.

The systematic sampling technique was employed to reach the study participants. The sample size was divided proportionally between the three kebeles. The total number of households having children aged 6–23 months in the town was taken from the town health office and then was divided by the total sample size to determine the interval between consecutive households having children aged 6–23 months, and sampling interval was calculated. Then, 517 households were selected using a systematic sampling method. The first household was randomly selected and the next households were selected based on the interval. For households with more than one child aged 6–23 months, a child was selected using the lottery method. When the mother/caregiver was not available at the time of data collection, another one-time revisit was made on the same day. When that fails, the nearby household having the target child was considered.

### 2.5. Data Collection Procedures

Data were collected using an interviewer-administered and structured questionnaire. A questionnaire was prepared in English and translated into the local language (Afan Oromo) and then translated back to English. Pretest was conducted on 5% of households before actual data collection. Necessary revisions were done based on the gaps identified during the pretest interview. The questionnaire was designed to ask about their practice first before asking them on their knowledge. This was to ensure that the participants' responses to their practices would not be biased by the questions on knowledge.

The data collection tools regarding the various socioeconomic and demographic variables were adopted from EDHS questionnaire with some modification to fit with the context. Data collection tool for dietary diversity practices were adapted from FAO guidelines for assessing nutrition-related KAP and WHO indicators for assessing IYCF practices [6, 27]. Food lists were adapted to reflect locally available foods and then translated into local languages before questioning about food consumption practices.

The recall period of 24 hours' method of assessing dietary diversity practice was used. Interviewers have visited the households and asked mothers what their child had consumed in the past 24 hours. Supervisors and data collectors were trained on the overall introduction of the study process, including its objective and technique of interviewing, and on definitions of terms used in the questionnaire. The collected data were checked every day by the investigator and correction feedback was given every morning for supervisors and data collectors.

### 2.6. Data Analysis

Data were entered in EpiData and exported to SPSS version 20 for further analysis. Frequency and percentages of each variable were calculated and displayed using tables. Descriptive measures for continuous variables were calculated and their normality distributions were checked. Bivariate logistic regression was used to check which variables had an association with the dependent variable individually and multivariate logistic regression was conducted to identify factors that were associated with minimum dietary diversity practice. All variables with a *P* value <0.2 in the bivariate logistic regression were moved into multivariate logistic regression. Then, independent factors were determined to be associated with outcome variable using *P* value ≤0.05. Adjusted odds ratios with 95% confidence interval were used to assess the strength of association and level of significance.

### 2.7. Study Variables

#### 2.7.1. Dependent Variable

Minimum dietary diversity practice was the outcome variable of the study. Minimum dietary diversity score was calculated by summing the number of food groups consumed by the individual child over the 24-hour recall period. Then, depending on the WHO's standardized cutoff point, it was transformed to binary variable (adequate or inadequate dietary diversity).

#### 2.7.2. Independent Variables

Parental-level (maternal age, maternal education, maternal knowledge, maternal occupation, marital status of the mother, place of delivery, religion, ethnicity, paternal education, and paternal occupation), child-level (age of the child, sex, birth order of index child, growth monitoring followup, and breastfeeding status) and household-level variables (exposure to media, number of under-five aged children, family size, and monthly income) were considered as independent variables.

### 2.8. Operational Definitions

#### 2.8.1. Minimum Dietary Diversity Practice

Proportion of children aged 6–23 months receive four or more out of seven food groups. The seven food groups used to create the score of this indicator are grains, roots, and tubers; legumes and nuts; dairy products (milk, yogurt, and cheese); flesh foods (meat, fish, poultry, and liver/organ meats); eggs; vitamin A rich fruits and vegetables; other fruits and vegetables [6].

#### 2.8.2. Knowledge on Diet Diversification

To assess the level of knowledge on diet diversification, mothers were asked one question that has a list of seven correct answers. Each respondent is given a score based on the number of correct responses provided. Those mothers who had below the mean score were considered as not having knowledge on dietary diversification and those who had above the mean score were considered as having knowledge on dietary diversification [27].

#### 2.8.3. Exposure to Media

A respondent reads newspaper, watches television, or listens to the radio at least once a week [20].

## 3. Results

### 3.1. Socioeconomic and Demographic Characteristics

A total of 508 mothers having children aged 6–23 months participated in the study, making a response rate of 98%. One hundred ninety-nine (39.2%) respondents were in the age group 25–29 years, 337 (66.3%) were Muslims, and 470 (92.5%) were Oromos. Concerning the educational status of the mothers, 61 (12%) had no formal education and three fourth of the participating mothers were housewives. Forty five (9%) fathers had no formal education, and 180 (35.4%) were daily laborers.

### 3.2. Household and Child-Level Characteristics

Regarding households of the study participants, 293 (57.7%) households had only one under-five children and 261 (51.4%) households had a total of family members of five and above. Almost all mothers were involved in deciding the type of food to provide for their child at the household level, and 153 (30%) mothers did not have media exposure. From the total children included, 353 (69.5%) were in the age group 12–23 months. Four hundred seventy-two (92.9%) children were born at health institutions (hospital or health center). The majority of children (446; 87.8%) conducted growth monitoring followup (Table 1).

### 3.3. Types of Food Groups Consumed

Cereals and tubers were the most consumed food groups among the seven food groups in 24 hours prior to the data collection day. The majority of mothers (480; 94.5%) responded that their children ate foods prepared from grains, roots, and tubers, whereas flesh foods (meat, poultry, and fish products),and eggs were the less consumed food groups. Fifty four (11%) and 133 (26%) children ate flesh foods and eggs, respectively (Table 2).

### 3.4. Knowledge on Food Diversification

Knowledge of mothers on food diversification is assessed by asking them which type of food they use or ways to make food more nutritious or better for their child's health.

**Figure 1:**
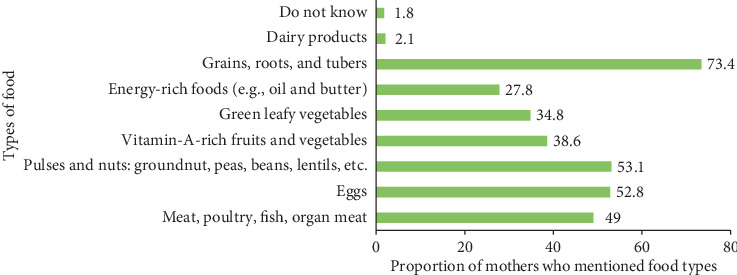
Proportion of mothers mentioning types of foods they use to make their child's diet more nutritious or diversified.

Two hundred eighty-four (56%) respondents mentioned three and more (>mean score) food types to enrich the complementary foods. Nine (1.8%) mothers responded that they do not know which types of foods they use to make the diet more nutritious (Figure 1).

### 3.5. Minimum Dietary Diversity Practice

According to mothers' report of what their children had consumed in the 24 hours prior to the day of the data collection, the mean dietary diversity score out of the seven food groups was 2.89 with SD of 1.17. The study revealed that 118 (23%) infants and young children consumed the required minimum number of food groups (≥4 food groups), and the rest (390; 77%) of infants and young children in the study area received below the minimum recommended dietary diversity.

### 3.6. Factors Associated with Dietary Diversity Practices

Of the variables evaluated in bivariate and multivariable logistic regression analysis, the child's age, mothers' education, media exposure, fathers' occupational status, and knowledge of mothers on food diversification were the only variables which have shown a statistically significant and independent association with minimum dietary diversity practices. The analysis indicated that children aged 12–23 months were more likely to have minimum dietary diversity compared with children aged 6–11 months (AOR = 2.99, 95% CI, 1.65 : 5.42). This study also indicated that children of mothers having secondary and above education level were more likely to be fed diversified food. It was shown that, among mothers whose education level was secondary or above, the odds of feeding their children minimum diversified diet were nearly three times higher than those mothers who have no formal education (AOR = 3.21, 95% CI, 1.05 : 9.85).

Mothers who were exposed to the media frequently had higher odds of feeding their children diversified diet than those mothers who had not attended to the media (AOR = 3.99, 95% CI, 1.97 : 7.77). The study showed that mothers aged 15–24 years were more likely to feed their child diversified food than older mothers. Children whose mothers aged 30 or above years were 56% less likely to meet the minimum dietary diversity score compared with children whose mothers aged 15–24 years (AOR = 0.44, 95% CI, 0.22 : 0.87). Mothers who have knowledge about food diversification showed significant association with their habit of dietary diversity feeding practice. Children whose mothers were knowledgeable about food diversification had 8.5 times higher odds of minimum dietary diversity practice compared with children whose mothers do not have specific knowledge on food diversification (AOR = 8.5, 95% CI, 4.95 : 14.58).

Children whose fathers were daily laborers showed a low practice of minimum dietary diversity compared with children whose fathers were merchants. Children whose fathers' were merchants were two times more likely to get a diversified diet than children whose fathers were daily laborers (AOR = 2.27, 95% CI, 1.19 : 4.33) (Table 3).

## 4. Discussion

Staple foods of the study area are bread and “injera,” which are mainly prepared from the main crops in the area (wheat, barley, and teff). According to the findings of this study, grains, roots, and tubers were found to be the most consumed food group. This finding comes similar to studies conducted in different part of Ethiopia [15, 28, 29]. This implies that infants and young children could get foods consisting mainly of carbohydrates, which provide energy but lacks enough protein, iron, and zinc [12].

Flesh foods (meat, poultry, and fish products) and eggs were the less consumed food groups in the current study area. This is a similar challenge in different parts of Ethiopia, Rwanda, and Burundi [14–17, 29, 30]. However, the consumption rate of legumes and nuts in the current study area was higher compared to the result of the study conducted at Arsi Negele [28], but lower than studies conducted in Dangila town of Northwest Ethiopia and Wolaita Sodo town of Southern Ethiopia [16, 31]. The difference might be a result of the difference in the study period and setting.

As the study revealed, 77% of children in the study area had below the minimum recommended dietary diversity for children aged 6–23 months. The rest of them consumed the minimum number of food groups required (≥4 food groups). This is parallel to the study done at Southern Ethiopia, Mozambique, and rural Rwada [15, 19, 30], but higher when compared to studies previously conducted at Arsi Negele, Gorche district, and Dangila town [25, 28]. However, this finding is lower than other similar studies done in Wolaita Sodo of Ethiopia (27.3%), Addis Abeba (59.9%), and Nepal of Asian country (30.4%) [23, 31, 32]. The reasons for the discrepancy and similarity might be associated with difference in socioeconomic status, study setting, sample size, and study period.

In the current study we used the dietary diversity standard developed previously which is described in WHO 2010 document [6]. It describes seven food groups. Based on the June 2017 expert consultation, breast milk has been added as an eighth food group, and the criterion for MDD has shifted, accordingly, from four of seven groups to five of eight groups [33]. This might slightly change the current findings if analysed by using the new modified indicator.

Concerning factors associated with minimum dietary diversity practice there are few studies which found infants aged 6–11 months receiving adequate dietary diversity compared with older children [14, 15]. The current study, however, revealed that children aged 12–23 months receive adequate dietary diversity compared with infants. This is consistent with several studies conducted before. For instance, studies conducted in Dangila town [16], Gorche district [25], evaluation study in rural Ethiopia [26], and analysis of 2016 Nepal Demographic and Health Survey [34] came up with similar finding. This result might be because most mothers do not introduce cereals and legumes for very young children aged 6–11 months until they are one year of age [34]. Further analyses of the current study suggest that one reason for poor dietary diversity among infants is delayed initiation of complementary food. Ten percent of the total participants, all aged less than one year, did not consume any type of food other than milk.

The study also showed the educational status of mothers was one of the significant factors that determine if mothers fed their children an adequately diversified diet. Educated mothers were more likely to feed their children diversified food compared with mothers who received no formal education. Studies from Ethiopia and Nepal came up with a parallel finding [14, 22, 23]. This could be because educated mothers are more expected to know locally available nutritious foods and have higher ability to capture nutritional messages (information) through different mass media.

Mothers who had been exposed to different media like television and radio at least once a week or frequently were more likely to provide their children an adequately diversified diet compared with those who had not been exposed to media of any kind. This finding is similar to a study done in Dangila town and the analysis of Ethiopian Demographic and Health Survey [16, 22]. The possible reason could be that the dissemination of child nutrition-related information through media in national radio and television has a positive influence on mothers' feeding practices, due to the fact that their nutritional knowledge could increase through regular exposure.

The study identified that younger mothers were two times more likely to feed their child diversified food than older mothers. It was similar to the finding of the analysis of Ethiopian Demographic and Health Survey that showed older women were giving their children less diverse diets [17]. However, it was different from the study conducted in Nepal [23]. The possible justification might be that the older mothers might have several children in the household. These mothers could not give their children diversified diet since it is more difficult to support a large family. However, this finding needs another supportive study for justification.

Maternal knowledge of food diversification showed strong positive associations with dietary diversity feeding practice. It was consistent with the study conducted in Adea district [35]. The possible explanation for this finding is that, even if families can afford different food varieties which are available from the local market, the awareness of mothers on the nutritional value of the different variety of foods is a crucial factor to purchase these foods regularly for their children. The main problem identified in the current study was that mothers perceive foods of the same nutritional value as having different functions for children's health. Most of the mothers perceived that different products of cereals, which are in the same food group, such as pasta, rice, and macaroni, can provide different sources of nutrient for their children. This affects the growth and development of their children.

## 5. Limitations

The study is not free of recall bias and social desirability bias. It may not also accurately reflect the children's past feeding experience since it considers only 24-hour feeding.

## 6. Conclusion

In general, infants and young children in the current study area who receive minimum dietary diversity were scarce. A large proportion of mothers are feeding their children only grain, roots, and tubers or dairy products due to their limited knowledge on diet diversification and other factors such as child's age, mothers' education, media exposure, and fathers' occupational status.

It is recommended that healthcare workers counsel mothers on how they can increase food variety, especially mothers of infants under one year of age. Moreover, mother-targeted health education programs, focusing on knowledge on nutritional values of locally available foods and nutritious foods which are needed for infants and young children's health and development, are important.

The health sector needs to strengthen broadcasting information through available local media on complementary food diversification with locally available foods. Other sectors like the educational sector require promoting female education, as they become mothers in the future and also educated mothers tend to feed their children diversified food.

## Figures and Tables

**Table 1:** Parental and child characteristics of the study participants.

Variables	Frequency	Percentage
Maternal age		
15–19	14	2.8
20–24	153	30.1
25–29	199	39.2
30–34	84	16.5
≥35	58	11.4

Maternal education		
No education	61	12.0
Primary (grades 1–8)	245	48.2
Secondary and above	202	39.8

Paternal education		
No education	45	8.9
Primary (grades 1–8)	241	47.4
Secondary and above	222	43.7

Mother's occupation		
Merchant	75	14.8
Government employee	37	7.3
Housewife	374	73.6
Other^a^	22	4.4

Father's occupation		
Merchant	166	32.7
Employed	97	19.1
Labor worker	180	35.4
Farmer	55	10.8
Other^b^	10	2

Child sex		
Male	247	48.6
Female	261	51.4

Child age		
6–11	155	30.5
12–23	353	69.5

Place of delivery		
Health institution	472	92.9
Home	36	7.1

Birth order		
First	137	27.0
2–4	288	56.7
>4	83	16.3

Growth monitoring followup		
Yes	446	87.8
No	62	12.2

Currently breast feeding		
Yes	454	89.4
No	54	10.6

^a^Labor worker, farmers, and students. ^b^Drivers and students.

**Table 2:** Type of food children consumed during 24 hours prior to the day of data collection.

Variables (food groups)^*∗*^	Frequency	(%)
1. Grain, roots, and tubers	480	94.5
2. Legumes and nuts	207	40.7
3. Dairy products	363	71.5
4. Flesh foods (meat)	54	10.6
5. Eggs	133	26.2
6. Vitamin A rich fruits and vegetables	142	28.8
7. Other fruits or vegetables	87	17.1

^*∗*^The question has multiple responses.

**Table 3:** Bivariate and multivariate logistic regression analysis of factors on minimum dietary diversity practice.

Predictor variables	MDD score		COR (95% CI)	AOR (95% CI)
	Yes	No		
Child age (month)				
6–11	21 (14)	134 (86)	1	1
12–23	97 (28)	256 (72)	2.42(1.44, 4.05)^*∗∗*^	2.99 (1.65, 5.42)^*∗∗*^

Current breastfeeding status				
No	21 (39)	33 (61)	1	1
Yes	97 (21)	357 (79)	0.43 (0.24, 0.77)^*∗∗*^	0.62 (0.29, 1.31)

Place of delivery				
Home	5 (14)	31 (86)	1	1
Health facility	113 (24)	359 (76)	1.95 (0.74, 5.14)	1.32 (0.42, 4.16)

Marital status				
Not in union	1 (5)	21 (95)	1	1
In union	117 (24)	369 (76)	6.66 (0.89, 50.04)	2.10 (0.25, 17.82)

Mothers' decision				
No	1 (7)	13 (93)	1	1
Yes	117 (24)	377 (76)	4.03 (0.52, 31.17)	3.01 (0.28, 32.52)

Media exposure				
No	14 (9)	138 (90)	1	1
Yes	104 (29)	252 (71)	4.07 (2.24, 7.38)^*∗∗*^	3.91 (1.97, 7.77)^*∗∗*^

Mother's education				
No education	5 (8)	56(92)	1	1
1^◦^ education	44 (18)	201 (82)	2.45 (0.93, 6.48)	1.80 (0.60, 5.34)
2^◦^ and above	69 (34)	133 (66)	5.81 (2.22, 15.17)^*∗∗*^	3.21 (1.05, 9.85)^*∗*^

Maternal age				
15–24	49 (29)	118 (71)	1	1
25–29	49 (25)	150 (75)	0.79 (0.49, 1.25)	0.61 (0.34, 1.08)
≥30	20 (14)	122 (86)	0.39 (0.22, 0.70)^*∗∗*^	0.44 (0.22, 0.87)^*∗*^

Knowledge on food diversification				
Do not know	25 (9)	259 (91)	1	1
Knows	93 (42)	131 (58)	7.35 (4.51, 11.99)^*∗∗*^	8.50 (4.95, 14.58)^*∗∗*^

Father's education				
No education	6 (13)	39 (87)	1	1
1^◦^ education	43 (18)	198 (82)	1.41 (0.56, 3.54)	0.52 (0.18, 1.52)
2^◦^ and above	69 (31)	153 (69)	2.93 (1.19, 7.25)^*∗*^	0.69 (0.23, 2.10)

Father's occupation				
Daily laborer	25 (14)	154 (86)	1	1
Employed	35 (36)	62 (64)	3.48 (1.92, 6.28)^*∗∗*^	2.14 (0.99, 4.59)
Merchants	44 (26)	123 (74)	2.20 (1.28, 3.80)^*∗∗*^	2.27 (1.19, 4.33)^*∗*^
Others	14 (22)	51 (79)	1.69 (0.82, 3.50)	2.41 (0.99,5.82)

No of children <5				
1	70 (24)	223 (76)	1	1
2	44 (24)	137 (76)	1.02 (0.66, 1.58)	1.03 (0.56, 1.90)
3 and above	4 (12)	30 (88)	0.42 (0.14, 1.25)	1.27 (0.36, 4.48)

Family size				
Less than 5	74 (30)	174 (70)	1	1
5 or more	44 (17)	216 (83)	0.48 (0.31, 0.73)^*∗∗*^	0.80 (0.45, 1.44)

^*∗∗*^Statistically significant at *P* < 0.01. ^*∗*^Statistically significant at *P* < 0.05.

## Data Availability

The data used to support the findings of this study have not been made available because there is no right to share it. All these findings are ongoing and hence cannot be shared.

## Abbreviations

AOR: Adjusted odds ratio
CI: Confidence interval
COR: Crude odds ratio
EDHS: Ethiopian Demographic and Health Survey
FAO: Food and agricultural organization
IYCF: Infant and young child feeding
KAP: Knowledge, attitude, and practices
MDD: Minimum dietary diversity
SPSS: Statistical Package for Social Sciences.

## References

[1] World Bank (2006). *Repositioning Nutrition as Central to Development: A Strategy For Large-Scale Action*.

[2] United Nations Children’s Fund, Improving Child Nutrition (2013). *The Achievable Imperative for Global Progress*.

[3] World Health Organization (2015). *Improving Nutrition Outcomes with Better Water. Sanitation and Hygiene: Practical Solutions for Policies and Programmes*.

[4] Rah J. H., Akhter N., Semba R. D. (2010). Low dietary diversity is a predictor of child stunting in rural Bangladesh. *European Journal of Clinical Nutrition*.

[5] Kimiywe J., Chege P. M. (2015). Complementary feeding practices and nutritional status of children 6-23 months in Kitui County, Kenya. *Journal of Applied Biosciences*.

[6] World Health Organization (2010). *Indicators for Assessing Infant and Young Child Feeding Practices Part 2 Measurement: 20 Avenue Appia*.

[7] Nti C.A. (2011). Dietary diversity is associated with nutrient intakes and nutritional status of children in Ghana. *Asian Journal of Medical Sciences*.

[8] Kennedy G. L., Pedro M. R., Seghieri C., Nantel G., Brouwer I. (2007). Dietary diversity score is a useful indicator of micronutrient intake in non-breast-feeding Filipino children. *Journal of Nutrition*.

[9] Khamis A. G., Mwanri A. W., Ntwenya J. E., Kreppel K. (2019). The influence of dietary diversity on the nutritional status of children between 6 and 23 months of age in Tanzania. *BMC Pediatrics*.

[10] Krasevec J., An X., Kumapley R., Bégin F., Frongillo E. A. (2017). Diet quality and risk of stunting among infants and young children in low and middle‐income countries. *Maternal & Child Nutrition*.

[11] Marriott B. P., White A., Hadden L., Davies J. C., Wallingford J. C. (2012). World health organization (WHO) infant and young child feeding indicators: association with growth measures in 14 low-income countries. *Maternal & Child Nutrition*.

[12] World Health Organization (2009). *Infant and Young Child Feeding Model Chapter for Textbooks for Medical Students and Allied Health Professionals*.

[13] Food and Agriculture Organization (2011). *Guidelines for Measuring Household and Individual Dietary Diversity, Nutrition and Consumer Protection Division*.

[14] Aemro M., Mesele M., Atenafu A., Birhanu Z. (2013). Dietary diversity and meal frequency practices among infant and young children aged 6–23 months in Ethiopia: a secondary analysis of Ethiopian demographic and health survey 2011. *Journal of Nutrition and Metabolism*.

[15] Gatahun E. A., Abyu D. M. (2015). Dietary diversity feeding practice and determinants among children aged 6-23 Months in kemba woreda, southern Ethiopia implication for public health intervention. *Journal of Nutrition and Food Sciences*.

[16] Beyene M., Gebeyehu A., Melese M. (2015). Dietary diversity, meal frequency and associated factors among infant and young children in Northwest Ethiopia: a cross-sectional study. *BMC Public Health*.

[17] Headey D. (2014). *An Analysis of Trends and Determinants of Child Undernutrition in Ethiopia, 2000–2011, ESSP II Working Paper 70*.

[18] Central Statistical Agency (Ethiopia) and ICF (2016). *Ethiopia Demographic And Health Survey 2016: Key Indicators Report: Addis Ababa, Ethiopia, and Rockville*.

[19] Yunhee K., Kudakwashe C., Joan M., Aashima G. (2019). Determinants of minimum dietary diversity among children aged 6–23 Months in 7 countries in East and southern africa (P10-035-19). *Current Developments in Nutrition*.

[20] Central Statistical Agency (Ethiopia) and ICF International (2012). *Ethiopia Demographic and Health Survey 2011*.

[21] Hirvonen K. (2016). *Rural-urban Differences in Children’s Dietary Diversity in Ethiopia: A Poisson Decomposition Analysis. ESSP II Working Paper 89*.

[22] Disha A., Tharaney M., Abebe Y., Alayon S., Winnard K. (2015). *Factors Associated with Infant and Young Child Feeding Practices in Amhara Region and Nationally in Ethiopia: Analysis of the 2005 and 2011 Demographic and Health Surveys*.

[23] Khanal V., Sauer K., Zhao Y. (2013). Determinants of complementary feeding practices among Nepalese children aged 6–23 months: findings from demographic and health survey 2011. *BMC Pediatrics*.

[24] Iqbal S., Zakar R., Zakar M. Z., Fischer F. (2017). Factors associated with infants’ and young children’s (6–23 months) dietary diversity in Pakistan: evidence from the demographic and health survey 2012–13. *Nutrition Journal*.

[25] Dangura D., Gebremedhin S. (2017). Dietary diversity and associated factors among children 6-23 months of age in Gorche district, Southern Ethiopia: cross-sectional study. *BMC Pediatrics*.

[26] Kuche D., Moss C., Eshetu S., Ayana G. (2020). Factors associated with dietary diversity and length‐for‐age z‐score in rural Ethiopian children aged 6–23 months: a novel approach to the analysis of baseline data from the sustainable undernutrition reduction in Ethiopia evaluation. *Maternal & Child Nutrition*.

[27] Macías Y. F., Glasauer P. (2014). *Guidelines for Assessing Nutrition-Related Knowledge, Attitudes and Practices-KAP Manual*.

[28] Kassa T., Meshesha B., Haji Y., Ebrahim J. (2016). Appropriate complementary feeding practices and associated factors among mothers of children age 6–23 months in Southern Ethiopia. *BMC Pediatrics*.

[29] Temesgen H., Yeneabat T., Teshome M. (2018). Dietary diversity and associated factors among children aged 6–23 months in sinan woreda, Northwest Ethiopia: a cross-sectional study. *BMC Nutrition*.

[30] Estefania C., Zaida H., Tharcisse N., Dorota W. (2019). Children’s dietary diversity and related factors in rwanda and burundi: a multilevel analysis using 2010 demographic and health surveys. *PLoS ONE*.

[31] Mekonnen T. C., Workie S. B., Yimer T. M., Mersha W. F. (2017). Meal frequency and dietary diversity feeding practices among children 6–23 months of age in Wolaita Sodo town, Southern Ethiopia. *Journal of Health, Population and Nutrition*.

[32] Dagmawit S., Zewdie A., Tegegne T. K (2017). Minimum dietary diversity and associated factors among children aged 6-23 months in Addis Ababa, Ethiopia. *International Journal for Equity in Health*.

[33] World Health Organization (2017). *Global Nutrition Monitoring Framework: Operational Guidance for Tracking Progress in Meeting Targets for 2025*.

[34] Baek Y., Chitekwe S. (2019). Sociodemographic factors associated with inadequate food group consumption and dietary diversity among infants and young children in Nepal. *PLoS ONE*.

[35] Agize A., Jara D., Dejenu G. (2017). Level of knowledge and practice of mothers on minimum dietary diversity practices and associated factors for 6–23-month-old children in Adea woreda, Oromia, Ethiopia. *BioMed Research International*.

